# Enhancing Communication Skills of Individuals With Autism Spectrum Disorders While Maintaining Social Distancing Using Two Tele-Operated Robots

**DOI:** 10.3389/fpsyt.2020.598688

**Published:** 2021-01-25

**Authors:** Hirokazu Kumazaki, Taro Muramatsu, Yuichiro Yoshikawa, Hideyuki Haraguchi, Taichi Sono, Yoshio Matsumoto, Hiroshi Ishiguro, Mitsuru Kikuchi, Tomiki Sumiyoshi, Masaru Mimura

**Affiliations:** ^1^Department of Preventive Intervention for Psychiatric Disorders, National Center of Neurology and Psychiatry, National Institute of Mental Health, Tokyo, Japan; ^2^Department of Clinical Research on Social Recognition and Memory, Research Center for Child Mental Development, Kanazawa University, Ishikawa, Japan; ^3^Department of Neuropsychiatry, Keio University School of Medicine, Tokyo, Japan; ^4^Department of Systems Innovation, Graduate School of Engineering Science, Osaka University, Osaka, Japan; ^5^JST ERATO ISHIGURO Symbiotic Human-Robot Interaction, Osaka, Japan; ^6^Service Robotics Research Group, Intelligent Systems Institute, National Institute of Advanced Industrial Science and Technology, Ibaraki, Japan

**Keywords:** autism spectrum disorders, COVID-19, social distancing, robot, communication skill, motivation

## Abstract

COVID-19 has affected many areas of daily life, including communication and learning. Social distancing is essential to prevent the spread of COVID-19. In these situations, teaching communication skills is essential for helping individuals with autism spectrum disorders (ASD) reach their full potential. To provide communication education while maintaining social distancing, we developed a communication training system using a tele-operated robot. In this system, we prepared a PC and a robot for each participant. The participants were grouped in pairs and communicated with each other through the tele-operated robot. The objective of this study was to test whether this system can maintain motivation for training in individuals with ASD and whether our system was useful for improving communication skills. Participants were randomly assigned to one of two groups: the taking a class by teachers alone (TCT) group or robot-mediated communication exercise (RMC) group. Participants in the TCT group took a class about communication skills from their teacher. Participants in the RMC group, in addition to taking a class by teacher, were grouped in pairs and communicated with each other through the tele-operated robot once a week over 4 weeks (for a total of five sessions). In total, twenty individuals with ASD participated in the study. One-way ANOVA revealed that there were significantly greater improvements in being good at describing their thoughts to others, which was self-rated (*F* = 6.583; *p* = 0.019), and good at listening to the thoughts or feelings of others, which was rated by themselves (*F* = 5.635; *p* = 0.029) and their teacher (*F* = 5.333; *p* = 0.033). As expected, the motivation for training using this system was maintained during a session. Overall, this study revealed that our system was useful for improving communication skills (e.g., listening to the thoughts or feelings of others). Teaching communication skills under pandemic conditions is important, and this study demonstrated the feasibility of communication training using tele-operated robots.

## Introduction

Autism spectrum disorder (ASD) is a range of conditions categorized by challenges with social skills, repetitive behaviors, speech and nonverbal communication (1). According to the Centers for Disease Control and Prevention (CDC), approximately one in 59 individuals in the United States are on the autism spectrum (2). Individuals with ASD have difficulty developing dialogue skills and understanding what others say to them. Teaching communication skills is essential for helping these individuals reach their full potential.

COVID-19 has affected many areas of daily life, including communication and learning. To prevent the spread of COVID-19, we cannot shake hands or touch each other. We have to wear a mask in public spaces and minimize social contact. Above all, social distancing is essential to prevent the spread of COVID-19. In these situations, to build and maintain social relationships, conveying thoughts to others and listening to the thoughts and feelings of others is important.

According to the “Intense World Theory” (3), individuals with ASD might perceive their surroundings not only as overwhelmingly intense due to hyper-reactivity of primary sensory areas but also as aversive and highly stressful due to an overly reactive amygdala. Therefore, they try to cope with the intense and aversive world by avoidance. According to “The Social Motivation Theory of Autism” (4), individuals with ASD can be construed as extreme cases of diminished social motivation. Social motivation is a powerful force guiding human behavior. It can be described as a set of psychological dispositions and biological mechanisms biasing individuals to preferentially orient to the social world (social orienting), seek and take pleasure in social interactions (social reward), and work to foster and maintain social bonds (social maintaining). Social motivation enables individuals with ASD to foster smooth relationships and promote coordination. Social communication intervention approaches are effective when they involve motivating activities and settings (5).

Unlike human beings, robots operate within predictable and lawful systems and thus offer individuals with ASD a highly structured learning environment that can help them focus on relevant stimuli. They have a higher degree of task engagement while communicating with robots than with human trainees and exhibit social behaviors toward robots (6). A growing body of literature has suggested that individuals with ASD have intrinsic motivation during interactions with robotic and technological systems (7–12). Furthermore, using robots can help us with social distancing. Robots can provide consistent and continuous support even during the COVID-19 pandemic because they have the advantage of providing opportunities for these individuals to engage without increasing their risk of infection. Thus, expectations for social robotics in supporting individuals with ASD seem higher than before.

**Figure 1:**
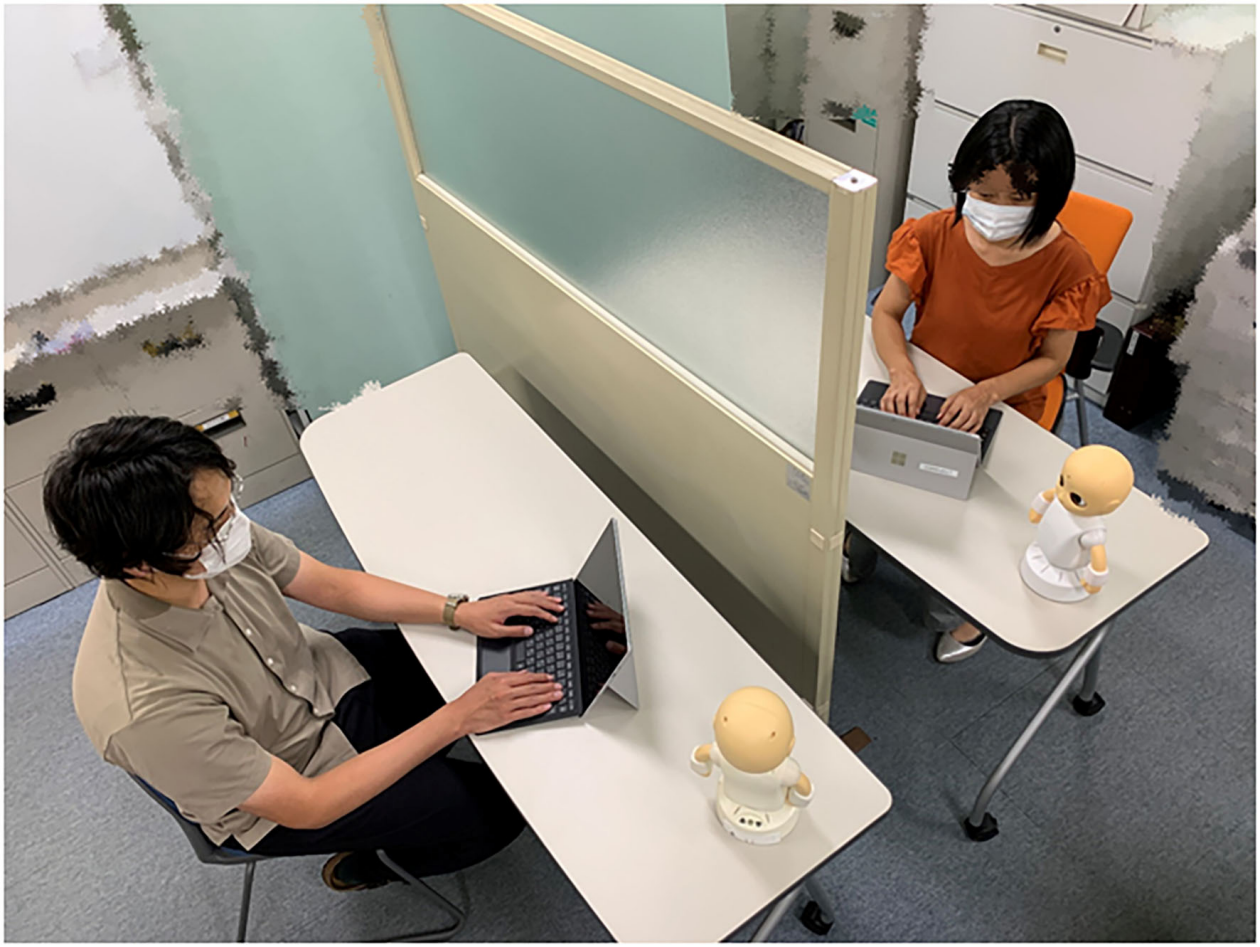
Communication training system using a tele-operated robot. In this system, two participants were in the same room. We prepared a PC and a robot for each participant. Participants were grouped in pairs and communicated with each other through the tele-operated robot. For example, one participant input words into the computer, which were read aloud by the CommU in front of them.

**Figure 2:**
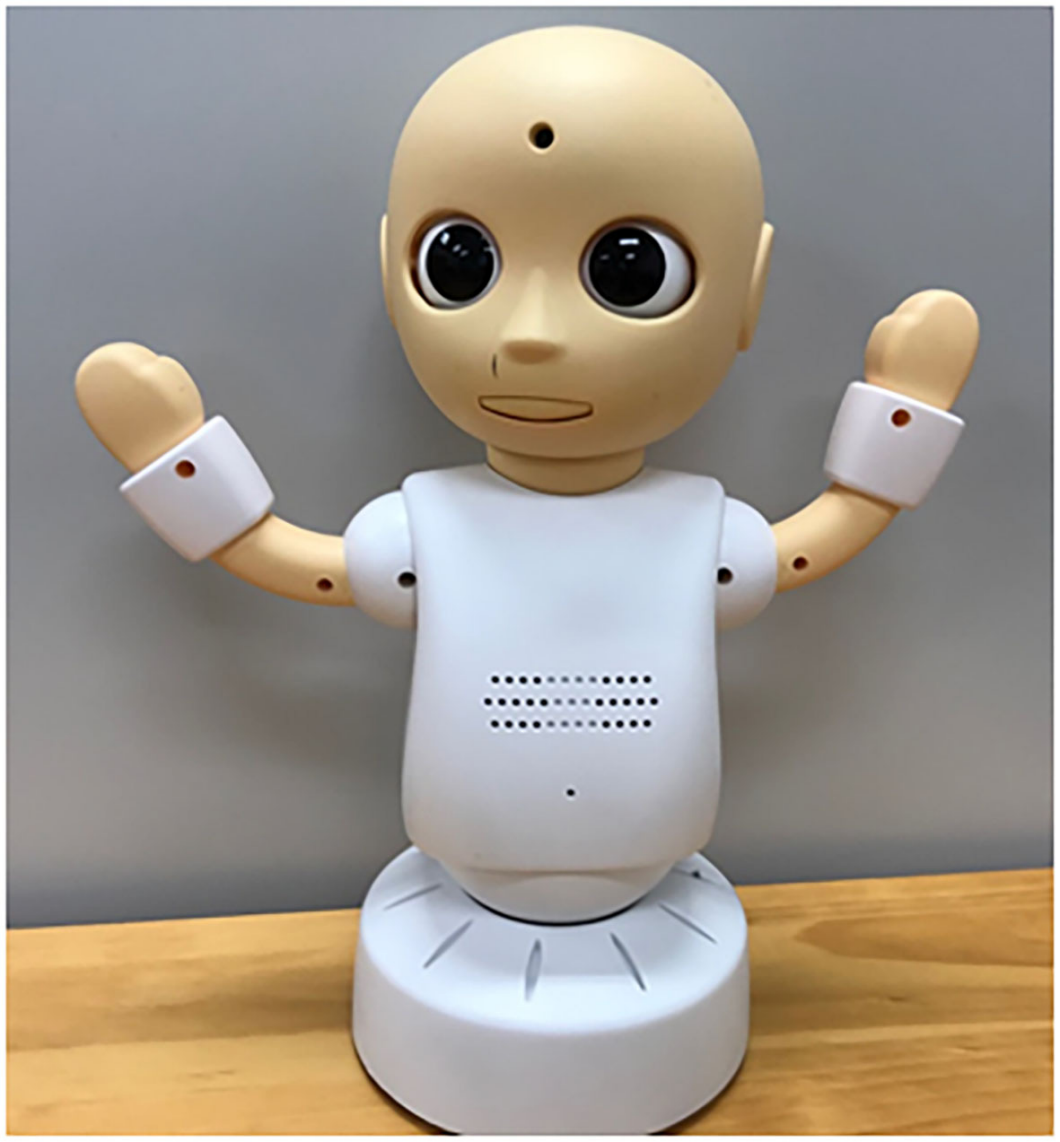
The CommU robot.

To provide communication education while maintaining social distancing, we developed robot-mediated communication exercise (RMC) using tele-operated robots. We selected robots rather than avatars because we considered a three-dimensional learning environment wherein a participant interacting with robots is more powerful than one in which the interaction is with avatars. In this system, two participants were in the same room. We prepared a PC and a robot for each participant. Participants were grouped in pairs and communicated with each other through the tele-operated robot (see Figure 1). The PCs that controlled each robot were placed in front of each participant. There is a divider between participants so that they do not need to look at each other and they can concentrate on the training. During the intervention, participants input words into the computer, which were read aloud by the robot CommU (see Figure 2). The participants could also replicate nonverbal expressions, such as nodding and lifting their hands with CommU. They could monitor the expressions made by the CommU controlled by the other participant whenever they wanted to see. In this experimental setup they were not allowed to speak aloud. The objective of this study was to test whether motivation for training using this system was maintained in individuals with ASD and whether our system was useful for improving communication skills (i.e., describing their thoughts to others and listening to the thoughts and feelings of others).

## Materials and Methods

### Participants

The present study was approved by the ethics committee of Kanazawa University. After receiving a complete explanation of the study, all participants and their guardians agreed to participate in the study. Written informed consent was obtained from the individuals and/or minors' legal guardian for the publication of any potentially identifiable images or data included in this article. All participants provided written informed consent. The inclusion criteria included (1) having a diagnosis of ASD based on the Diagnostic and Statistical Manual of Mental Disorders, Fifth Edition (DSM-5) from the supervising study psychiatrist (1), (2) being aged 15–22 years, and (3) having some experience of touch typing. (4) In addition, despite previous treatments such as social-skill training and behavioral therapy to improve communication skills, all participants were not good at describing their thoughts to others and listening to the thoughts and feelings of others. All participants had known each other for at least 1 year. At the time of enrollment, the diagnoses of all participants were confirmed by a psychiatrist with more than 10 years of experience in ASD using the criteria in the DSM-5 (1) and standardized criteria taken from the Diagnostic Interview for Social and Communication Disorders (DISCO) (13). The DISCO has been reported to have good psychometric properties (14).

All participants completed the Autism Spectrum Quotient-Japanese version (AQ-J) (15), which was used in the evaluation of ASD-specific behaviors and symptoms. The AQ-J is a short questionnaire with five subscales (social skills, attention switching, attention to detail, imagination, and communication). Previous work with the AQ-J has been replicated across cultures (16) and ages (17, 18). The AQ is sensitive to the broader autism phenotype (19). Full-scale IQ scores were measured by either the Wechsler Intelligence Scale for Children – Fourth Edition or the Wechsler Adult Intelligence Scale–Third Edition.

The severity of social anxiety symptoms was measured using the Liebowitz Social Anxiety Scale (LSAS) (20). This clinician-administered scale consists of 24 items, including 13 items that describe performance situations and 11 items that describe social interaction situations. Each item was separately rated for “fear” and “avoidance” using a 4-point categorical scale. According to receiver operating curve analyses, an LSAS score of 30 is correlated with minimal symptoms and is the best cutoff value for distinguishing individuals with and without social anxiety disorder (21).

The ADHD-RS (22) contains 18 items related to inattentive and hyperactive-impulsive symptoms, scored on a 4-point scale (0 = never, 1 = sometimes, 2 = often, 3 = very often), and assesses symptom severity over the past week. The total score was computed as the sum of the scores of all 18 items.

The CARS-TV is the Japanese version of the CARS (23)—one of the most widely used scales to evaluate the degree and profiles of autism in children—and has satisfactory reliability and validity (24). The CARS-TV consists of 15 items (“Relating to People,” “Imitation,” “Emotional Response,” “Body Use,” “Object Use,” “Adaptation to Change,” “Visual Response,” “Listening Response,” “Taste, Smell, and Touch Response and Use,” “Fear or Nervousness,” “Verbal Communication,” “Non-Verbal Communication,” “Activity Level,” “Level and Consistency of Intellectual Functioning,” and “General Impression”), each scoring from 1.0 (normal) to 4.0 (severely abnormal) in units of 0.5. The CARS-TV score is the sum of the scores of all 15 items, so that it can range from 15.0 to 60.0. In this study, experienced psychologists rated the subjects based on behavioral observation and parental reports.

### Procedures

The tele-operated robot used in this study was the CommU (25–27) (Vstone Co., Ltd.) which is 304 mm tall. CommU has 14 degrees of freedom (DoFs) as follows: waist (2), left shoulder (2), right shoulder (2), neck (3), eyes (3), eyelids (1), and lips (1). The careful design of the eyes and multiple DoFs dedicated to controlling its field of vision contribute to its rich gaze expressions. Its face can show a range of simplified expressions that are less complex than those of a real human face. The robot's cute shape, which resembles a child, is expected to be easy to anthropomorphize. Furthermore, its small and cute appearance is expected to help prevent fearfulness among individuals with ASD. In addition, CommU makes very little noise, and its controller is not distressed by its noise.

The participants were randomly assigned to two groups: taking a class by teachers alone (TCT) group or robot-mediated communication exercise (RMC) group. In the TCT group, the participants took a class about communication skills from their teacher. During these sessions, materials regarding the communication skills were provided. The participants were given access to feedback about it. The approximate duration of each class was 50 min per week. In the RMC group, in addition to taking a class by a teacher, the participants were grouped in pairs and communicated with each other through the tele-operated robot once a week over 4 weeks (for a total of five sessions). They practiced robot-mediated communication exercises about 15 min per session. We think that learning while concentrating is important. Given the low concentration span of individuals with ASD, we thought that a duration of 4 weeks and 15 min per session was appropriate. The participants started the conversation by selecting from the list of conversation topics (see Supplementary Material) that we prepared in advance. We decided which participants started the conversation, while they operated the robot in turns. If they could not carry on conversation about a particular topic, they moved on to the next topic. During the sessions, two digital video cameras were set up to capture the participants' performance.

Communication performance (i.e., good at describing their thoughts to others and listening to the thoughts and feelings of others) of all participants (i.e., TCT group and RMC group) in daily life was rated 2 weeks before and after the experiment by themselves and their teacher who stayed with and observed them for an hour or so every day. Emotions related to communication (i.e., describing my feelings and thoughts is fun, describing my feelings and thoughts is embarrassing, describing my feelings and thoughts is stressful, listening to others' feelings and thoughts is fun, listening to others' feelings and thoughts is embarrassing, listening to others' feelings and thoughts is stressful) in daily life were only self-rated before and after the experiment. These questionnaires were scored using a 7-point Likert scale. The ratings ranged from 1 (very poor) to 7 (very excellent) for communication performance. The ratings ranged from 1 (strongly disagree) to 7 (strongly agree) for emotions related to communication. The participants and their teacher attained a moderate degree of reliability [intraclass correlation coefficient (ICC) = 0.41] about the questionnaire (i.e., “good at describing the thoughts to others” and “good at listening to their thoughts and feelings of others”).

### Statistical Analysis

We performed the statistical analyses using SPSS version 24.0 (IBM, Armonk, NY, USA). Descriptive statistics for the sample were used. The differences in age and height, weight, IQ, AQ-J, LSAS-J, CARS total and subscale scores between the groups were analyzed using independent samples *t*-tests. The difference in gender proportion was analyzed using the χ^2^ test.

To investigate the difference in improvements between the two groups (i.e., TCT group and RMC group), one-way ANOVA with one group factor was performed to analyze the communication performance (i.e., good at describing their thoughts to others, good at listening to the thoughts and feelings of others) that were rated by their teacher and by themselves using baseline data as covariates. To investigate the differences in changes between the two groups, one-way ANOVA with one group factor was performed to analyze the emotions related to communication (i.e., describing my feelings and thoughts is fun, describing my feelings and thoughts is embarrassing, describing my feelings and thoughts is stressful, listening to others' feelings and thoughts is fun, listening to others' feelings and thoughts is embarrassing, listening to others' feelings and thoughts is stressful) using baseline data and AQ score as covariates.

Pearson product-moment correlation coefficient was used to explore the relationships between age, IQ, AQ, the total score of LSAS and ADHD-RS, verbal and non-verbal communication subscores of CARS, CARS total score, and the number of conversation turns and mental state terms. We employed an alpha level of 0.05 for these analyses.

## Results

### Feasibility and Participation

In total, twenty individuals with ASD participated in the study. All participants were Japanese. The RMC group included 10 participants (8 males) with a mean age of 19.8 ± 2.2 years. Participants in the RMC group were 169.5 ± 3.8 cm tall and weighed 69.0 ± 6.0 kg. The IQ score for the RMC group was 87.4 ± 15.0, their average AQ-J score was 33.2 ± 4.5, their LSAS-J score was 44.0 ± 7.4, their total ADHD-RS score was 13.4 ± 3.5, and their total CARS score was 33.7 ± 4.5. According to the CARS score, the autistic trait in the RMC group was mild in seven participants, moderate in one, and severe in two. The TCT group included 10 participants (9 males) with a mean age of 20.1 ± 2.4 years. The RMC group was 172.0 ± 4.3 cm tall and weighed 71.7 ± 5.1 kg. The IQ score for the TCT group was 84.8 ± 15.0, their average AQ-J score was 32.8 ± 3.6, their LSAS-J score was 40.7 ± 6.8, their total ADHD-RS score was 15.3 ± 5.0, and their total CARS score was 32.3 ± 1.8. There were no significant differences between groups with regard to mean ages (*p* = 0.77), gender proportion (*p* = 0.53), and IQ (*p* = 0.81), AQ-J (*p* = 0.83), LSAS-J (*p* = 0.31) scores, ADHD total score (*p* = 0.34), CARS total scores (*p* = 0.37). According to the CARS score, the autistic trait in the TCT group was mild in seven participants and moderate in three. Details are presented in Table 1.

**Table 1:** Descriptive Statistics of Participants in the RMC group and TCT group.

**Characteristics**	**RMC group (n=10), M (SD)**	**TCT group (n=10), M (SD)**	**Statistics**		
			***t* or χ^2^**	***df***	***p***
Age in years	19.8 (2.2)	20.1 (2.4)	*t* = −0.296	18	0.77
Gender (Male:Female)	8:2	9:1	χ^2^=0.392	1	0.53
Height	169.5 (3.8)	172.0 (4.3)	*t* = −1.368	18	0.19
Weight	69.0 (6.0)	71.7 (5.1)	*t* = −1.082	18	0.29
**Race (ratio)**					
Japanese	10/10	10/10			1.00
Full-scale IQ	87.4 (15.0)	84.8 (15.0)	*t* = −0.387	18	0.81
AQ-J	33.2 (4.5)	32.8 (3.6)	*t* = 0.219	18	0.83
LSAS-J	44.0 (7.4)	40.7 (6.8)	*t* = 1.038	18	0.31
ADHD-RS	13.4 (3.5)	15.3 (5.0)	*t*= −0.089	18	0.34
**CARS**					
1. Relating to people	2.4 (0.2)	2.3 (0.3)	*t* = 0.447	18	0.66
2. Imitation	1.5 (0.7)	1.1 (0.2)	*t* = 1.544	18	0.14
3. Emotional response	2.4 (0.4)	2.4 (0.4)	*t* = 0.000	18	1.00
4. Body use	2.2 (0.5)	2.3 (0.4)	*t*= −0.246	18	0.81
5. Object use	2.0 (0.4)	2.1 (0.7)	*t*= −0.629	18	0.54
6. Adaptation to change	2.4 (0.7)	2.2 (0.5)	*t*= 0.744	18	0.47
7. Visual response	2.4 (0.5)	2.1 (0.6)	*t*= 1.187	18	0.25
8. Listening response	2.3 (0.6)	2.6 (0.5)	*t*= −1.481	18	0.16
9. Taste, smell, and touch response and use	2.3 (0.4)	2.3 (0.4)	*t*= 0.000	18	1.00
10. Fear or nervousness	2.4 (0.6)	2.2 (0.5)	*t*= 0.600	18	0.56
11. Verbal communication	2.5 (0.4)	2.3 (0.5)	*t* = 1.168	18	0.26
12.Nonverbal communication	1.9 (0.5)	1.9 (0.4)	*t* = 0.239	18	0.81
13. Activity level	2.4 (0.5)	2.2 (0.3)	*t*= 0.805	18	0.43
14. Level and consistency of intellectual response	2.6 (0.6)	2.1 (0.5)	*t* = 1.985	18	0.06
15. General impression	2.5 (0.4)	2.3 (0.4)	*t*= 1.124	18	0.28
Total	33.7 (4.5)	32.3 (1.8)	*t*= 0.916	18	0.37

*SD, standard deviation; RMC group, robot-mediated communication exercise group; TCT group, taking a class by teachers alone group; M, mean; SD, standard deviation; AQ-J, autism spectrum quotient, Japanese version. In the AQ-J, higher scores reflect a greater number of ASD-specific behaviors; LSAS, Liebowitz Social Anxiety Scale*.

All participants in the RMC group completed the trial procedures without technological challenges or notable participant distress that would lead to session termination. We carefully observed participant performance and confirmed that all participants were concentrating during the trials and highly motivated from the start to finish of the experiment. Participants experienced 7.7 (SD = 0.7) conversation turns and 6.7 (SD = 1.0) mental state terms in each session on average. We found a significantly negative relationship between AQ and the average mental state terms per session in the RMC group (*r* = −0.77, *p* = 0.09).

### Primary Analyses

#### Communication Performance

One-way ANOVA revealed that there was a significantly greater improvement in good at describing their thoughts to others, which was self-rated (*F* = 7.734; *p* = 0.015). On the other hand, no improvements were observed in good at describing their thoughts to others, which was rated by their teacher (*F* = 0.638; *p* = 0.435). One-way ANOVA revealed that there were significantly greater improvements in good at listening to the thoughts or feelings of others, which was rated by themselves (*F* = 5.394; *p* = 0.033) and their teacher (*F* = 5.054; *p* = 0.038). Details regarding the communication performance scores are presented in Table 2.

**Table 2:** Means and standard devitation of the mean of the RMC group and TCT group in communication performance at baseline and postintervention and interaction effects between the RMC and TCT groups on communication performance.

**Outcome**	**Group**	**Baseline (M, SD)**	**Post intervention (M, SD)**	**Statistics**		
				***t***	**F**	***p***
“Good at describing their thoughts to others,” rated by themselves.	RMC	2.70 (1.34)	4.40 (1.71)	−2.847		0.019^*^
	TCT	3.90 (1.79)	4.00 (1.63)	−0.557		0.591
Interaction effect					7.734	0.015^*^
“Good at describing their thoughts to others,” rated by their teacher.	RMC	4.70 (1.77)	4.70 (1.49)	0.000		1.000
	TCT	3.60 (2.17)	4.20 (1.40)	−0.970		0.357
Interaction effect					0.638	0.435
“Good at listening to the thoughts and feelings of others,” rated by themselves.	RMC	3.80 (1.48)	4.70 (1.42)	−2.077		0.068
	TCT	4.90 (1.20)	4.60 (1.08)	1.152		0.279
Interaction effect					5.394	0.033^*^
“Good at listening to the thoughts and feelings of others,” rated by their teacher.	RMC	2.90 (1.73)	3.30 (1.70)	−2.499		0.037^*^
	TCT	3.10 (2.18)	2.70 (1.70)	1.309		0.223
Interaction effect					5.054	0.038^*^

*SD, standard deviation; RMC, robot-mediated communication exercise; TCT, taking a class by teachers alone*.

* *p < 0.05*.

#### Emotions Related to Communication

One-way ANOVA revealed that there was a significantly greater change in describing my feelings and thoughts is fun (*F* = 8.600; *p* = 0.009). No change was observed in describing my feelings and thoughts is embarrassing (*F* = 0.285; *p* = 0.600), describing my feelings and thoughts is stressful (*F* = 3.398; *p* = 0.083), listening to others' feelings and thoughts is fun (*F* = 0.033; *p* = 0.857), listening to others' feelings and thoughts is embarrassing (*F* = 0.981; *p* = 0.336), listening to others' feelings and thoughts is stressful (*F* = 2.224; *p* = 0.154). Details regarding the emotions related to communication are presented in Table 3.

**Table 3:** Means and standard devitation of the mean of the RMC group and TCT group for emotions related to communication at baseline and postintervention and interaction effects between the RMC and TCT groups on emotions related to communication.

**Outcome**	**Group**	**Baseline**	**Postintervention**	**Statistics**		
		**(M, SEM)**	**(M, SEM)**	***t***	**F**	***p***
Describing my feelings and thoughts is fun	RMC	3.30 (1.57)	4.10 (2.03)	−2.058		0.070
	TCT	3.30 (1.57)	2.80 (1.14)	1.627		0.138
Interaction effect					8.600	0.009^**^
Describing my feelings and thoughts is embarrassing	RMC	2.70 (2.00)	2.40 (1.35)	0.502		0.627
	TCT	3.70 (1.83)	4.00 (1.63)	−0.322		0.755
Interaction effect					0.285	0.600
Describing my feelings and thoughts is stressful	RMC	4.30 (1.49)	3.60 (1.77)	1.049		0.322
	TCT	4.00 (1.76)	4.80 (1.55)	1.309		0.223
Interaction effect					3.398	0.083
Listening to other's feelings and thoughts is fun	RMC	2.90 (1.00)	2.90 (1.29)	0.000		1.000
	TCT	3.10 (1.45)	3.20 (1.62)	−0.429		0.678
Interaction effect					0.033	0.857
Listening to other's feelings and thoughts is embarrassing	RMC	5.10 (1.91)	4.10 (1.73)	1.627		0.138
	TCT	5.20 (1.55)	5.20 (1.23)	0.000		1.000
Interaction effect					0.981	0.336
Listening to other's feelings and thoughts is stressful	RMC	4.10 (2.03)	4.00 (1.25)	0.218		0.832
	TCT	4.40 (1.78)	5.50 (0.85)	−1.673		0.129
Interaction effect					2.224	0.154

*RMC, robot-mediated communication exercise; TCT, taking a class by teachers alone*.

** *p < 0.01*.

## Discussion

In this study, we developed and provided a communication training system using a tele-operated robot for individuals with ASD to provide communication education while maintaining social distancing. As expected, motivation for training using this system was maintained during a session. Overall, this study revealed that our system was useful for improving communication skills (e.g., listening to the thoughts or feelings of others). Teaching communication skills under pandemic conditions is important, and the study demonstrated the feasibility of communication training using tele-operated robots.

Through the experience of communication training using tele-operated robots, the participants in the RMC group came to believe that describing their feelings and thoughts was more fun than those in the TCT group. These results could explain why motivation for training using this system was maintained during a session.

In this study, based on questionnaires with the participants and their teachers, our intervention indicated improved listening to the thoughts or feelings of others. Considering the usefulness of double scoring by subjects and their teachers (28), our results with the questionnaire on communication performance (i.e., good at listening to the thoughts or feelings of others) are reliable. On the other hand, the result regarding describing their thoughts to others did not match between the participants and their teachers. It is possible that they were in a state in which their skills for describing their thoughts to others could not be improved from the perspective of others. However, the fact that they became confident about describing their thoughts to others is important. In addition, they came to think that describing their feelings was fun, which may be linked to improving these skills in the near future.

Previous studies have demonstrated that individuals with ASD express fewer and shorter emotional state self-disclosure statements in personal narratives (29–32). Therefore, it is natural that we found a significant negative relationship between AQ and the average mental state terms per session in this study.

In common conversation, it may be hard for individuals with ASD to concentrate on listening to others partly because they are distracted by others' expressions. For many of these individuals, sensory overstimulation is a serious problem (25), and the flood of social cues from others' expressions may be a primary cause for the inability to process social signals (26). On the other hand, in the system developed here, they could directly avoid information and easily watch both robots, which facilitates information processing for individuals with ASD. In addition, the participants could freely control their part of the conversation and nonverbal communication by typing on a keyboard, which is easier for them than speaking face-to-face, and reduce the burden of speaking and direct their energy toward information processing (26). Moreover, for all participants, an unfamiliar person is difficult to interact with and therefore, pairing up familiar people supported a smooth interaction.

There is increasing anecdotal evidence that individuals with ASD might have unique opportunities to use robots for help (7–12, 33, 34). In most of these studies, individuals with ASD faced the robot directly. There are only a few studies in which individuals with ASD operated robots and communicated with others through teleoperated robots. In one study (33), participants using teleoperated robots and seeing their expressions improved their understanding of the perspectives of others. In another study (34), participants acted as interviewers in job interview training by running a teleoperated robot. This helped them understand the interviewer's point of view. Given the result of these studies, the experience of operating robots presents individuals with ASD, unique opportunities to learn to understand the perspectives of others. While in these studies, one participant operated the robot and another faced it, both participants in our study operated the robot and communicated with each other through it. In such settings, both participants understood the perspectives of interlocutors, something they could not achieve otherwise. This might have helped improve their communication skills so that they could describe their thoughts to others, and could listen to what others think and feel.

We would like to acknowledge several limitations of our study. The first is the relatively small number of participants. Larger sample sizes are necessary to provide more meaningful data to evaluate the efficacy of communication training systems using tele-operated robots for individuals with ASD. In the field of ASD support, long-term perspectives, such as the idea that individuals with ASD can grow up to lead successful and independent lives, are especially important. We would like to advance research on using social robots for individuals with ASD while also addressing related concerns based on long-term perspectives. The ratings by the subjects and their teachers is useful (28). The ICC score was 0.41. Given that this score was calculated by comparing the subjective self-evaluation of the participants and the objective evaluation by the teacher, it is natural that the concordance rate would not be high. Future studies could measure not only questionnaire responses but also biological markers such as saliva cortisol.

In conclusion, as hypothesized, individuals with ASD improved listening to the thoughts or feelings of others by using tele-operated robots for individuals with ASD. In addition, they demonstrated higher self-confidence that they are good at describing their thoughts to others. Communication education that considers social distancing is important to prevent the spread of COVID-19. The current work provides preliminary support for a unique application of a robotic system (e.g., communication training system using tele-operated robot) to improve communication skills while maintaining social distancing. On the other hand, there has been a longstanding concern that the use of robots for individuals with ASD may cause them to become addicted to using robots. We would like to advance research on the use of social robots for individuals with ASD while also addressing related concerns based on long-term perspectives.

## Data Availability Statement

The raw data supporting the conclusions of this article will be made available by the authors, without undue reservation.

## Ethics Statement

Written informed consent was obtained from the individuals and/or minors' legal guardian for the publication of any potentially identifiable images or data included in this article.

## Author Contributions

HK designed the study, conducted the experiment, carried out the statistical analyses, analyzed and interpreted the data, and drafted the manuscript. TM, YY, YM, HI, MK, TSu, MM, HH, and TSo conceived of the study, participated in its design, assisted with data collection, scoring of behavioral measures, analyzed and interpreted the data, were involved in drafting the manuscript, and revised it critically for important intellectual content. MM was involved in approving the final version to be published. All authors read and approved the final manuscript.

## Conflict of Interest

The authors declare that the research was conducted in the absence of any commercial or financial relationships that could be construed as a potential conflict of interest.
