# Supplementary material for: Enhancing Communication Skills of Individuals With Autism Spectrum Disorders While Maintaining Social Distancing Using Two Tele-Operated Robots
Source: Front Psychiatry. 2021 Jan 25;11:598688. doi: 10.3389/fpsyt.2020.598688 (PMC7868394; doi:10.3389/fpsyt.2020.598688)
Supplement: Supplementary file 1 [file Data_Sheet_1.DOCX]

Supplementary Material

List of conversation topics

・Your hobby

・Good things you have experienced recently

・Bad things you have experienced recently

・Your friends

・Your school

・Studying

・Your future

・Your family

・Thoughts about relationships

・Thoughts about marriage

・What would you like to say about yourself?
